# Video of the Month: Pulsating Umbilicus in a Neonate with Left Ventricular Diverticulum

**DOI:** 10.1055/s-0044-1791569

**Published:** 2024-10-03

**Authors:** M. Moormann, M. Vollroth, M. Lacher, H. Stepan, D. Gräfe, U. Thome, S. Rützel, M. Weidenbach, I. Martynov, C. Pügge

**Affiliations:** 1 Department of Pediatric Surgery, Leipzig University, Leipzig, Germany; 2 Department of Cardiac Surgery, Heart Center, Leipzig University, Leipzig, Germany; 3 Department of Obstetrics, Leipzig University, Leipzig, Germany; 4 Department of Pediatric Radiology, Leipzig University, Leipzig, Germany; 5 Department of Neonatology, Leipzig University, Leipzig, Germany; 6 Department of Pediatric Cardiology, Heart Center, Leipzig University, Leipzig, Germany

**Keywords:** left ventricular diverticulum, pulsating umbilicus, pentalogy of Cantrell

## Abstract

Left ventricular diverticulum (LVD) is a rare malformation presenting in 0.05% of all congenital cardiac anomalies. It is associated with additional cardiac and extracardiac malformations. We report on a female neonate with prenatally diagnosed heterotaxia and dextrocardia who was born with a pulsating supraumbilical mass. Echocardiography revealed a diverticulum originating from the left ventricle, which was connected to the umbilicus. Magnetic resonance imaging confirmed an LVD without evidence of a diaphragmatic hernia on the day of life 9. The child underwent laparotomy/lower sternotomy, and the diverticulum and epigastric hernia were closed. The postoperative course was uneventful, and the girl was discharged on the 10th postoperative day. In a neonate with a pulsatile supraumbilical mass, the diagnosis of a congenital LVD should be taken into consideration. The treatment is straightforward and was successful in this single case.

## Introduction

Ventricular diverticulum is an extremely rare congenital cardiac malformation accounting for 0.05% of all cardiac anomalies. 1 2 It is a finger-like protrusion from the ventricular cavity including all of the three wall layers contracting synchronically to the committing ventricle. 3 About 80% of them originate from the left ventricle, mostly emerging from the apex, whereas only 10% belong to the right or even both ventricles. 3 4

Congenital left ventricular diverticulum (LVD) is associated with other abnormalities such as cardiac and extracardiac defects. Cardiac malformations include ventricular septal defects, tetralogy of Fallot, tricuspid atresia, and malposition of the heart such as meso- or dextrocardia. 3 4 The most common extracardiac defects are persistent left superior caval vein and hypoplasia of the left pulmonary artery. 3 LVD can also be part of pentalogy of Cantrell (POC), a syndrome including five different congenital thoracic and abdominal malformations including anterior diaphragmatic hernia, sternal cleft, ectopia cordis, omphalocele, and intracardiac defects. 3 5 We present a neonate with a pulsatile supraumbilical mass that turned out to be an LVD.

## Case Report



**Fig. 1:**
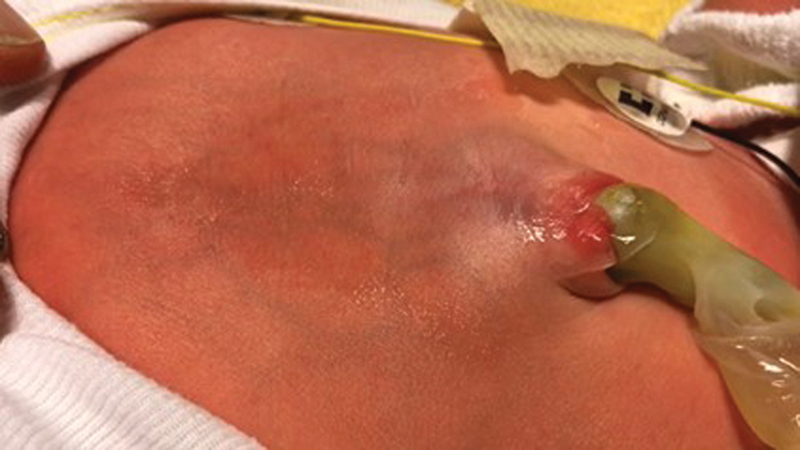
A 6-day-old neonate with a pulsating umbilical region contracting synchronically with the left ventricle.

**Video 1** :


A female neonate birth weight of 3,500 g was born at 40 weeks of gestation via vaginal delivery, with a pulsating supraumbilical mass ( Fig. 1 ).

**Fig. 2:**
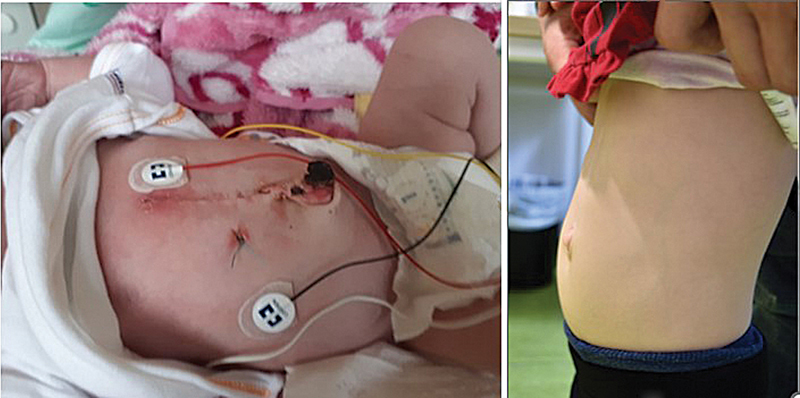
8 days and 2 years after surgery.

Prenatal sonography showed heterotaxia, a defect of left–right laterality with dextrocardia, and a ventricular septum defect. Except for a saturation of 85 to 90%, postnatal adaption was uneventful, and body measurements were within normal limits. On physical examination, the patient showed a pulsating midline structure between the umbilicus and the thorax which was covered by a thin layer of skin ( Video 1 ). Echocardiography revealed a persistent left superior vena cava, atrial and ventricular septum defect, a small persistent ductus arteriosus, and a mild tricuspid valve insufficiency without hemodynamic relevance. To further define the anatomy, a cardiac contrast-enhanced magnetic resonance imaging (MRI) was conducted. This showed a 1.0 × 0.8 cm LVD deriving from the left ventricle and a connection to the umbilicus with a total length of 3.7 cm ( Video 1 ). To prevent thrombosis of the diverticulum, anticoagulation with heparin was started. Surgery was performed on the seventh day of life by a pediatric and pediatric cardiac surgeon ( Video 1 ). Via median laparotomy and lower sternotomy, the pulsating diverticulum was exposed. Two clamps were placed distal to the base of the heart, and the diverticulum was divided. After closing both ends with polyproline sutures, the suture line was reinforced by a U-stitch including two pledgets. The distal part of the diverticulum was removed. As the underlying fascia of the abdominal wall between the umbilicus and sternum was absent, the epigastric hernia was closed with interrupted stitches. The postoperative course was uneventful. The girl was discharged 11 days after surgery. At 2 years follow-up, normal cardiac function was seen ( Fig. 2 ).

## Discussion

Since its first description by Friedrich Ludwig Kreysig in 1816, 809 cases of a ventricular diverticulum have been published. 6 An LVD is defined as a congenital outpouching that contains all three layers of the heart including the endocardium, myocardium, and pericardium. LVD differs from a left ventricular aneurysm (LVA) as it contracts synchronically with the left ventricle. Most often, it is an acquired condition with the absence of a muscular layer. 7 8 9 In our patient, clinical and diagnostic criteria lead to the diagnosis of LVD.

### Prenatal Diagnosis

Prenatal diagnosis of LVD is difficult because visualization of the left ventricular apex on ultrasonography is challenging. 4 Thus, only 2.4% of all LVD cases can be detected prenatally. 10 11 In our case, heterotaxia with dextrocardia and a ventricular septum defect without evidence of LVD were diagnosed prenatally. The main complications associated with LVD during pregnancy include rupture (38%), hydrops fetalis (19%), cerebral embolism, and arrhythmia. 10 12 In our case, no prenatal complications were encountered.

### Mode of Delivery

There are no data on the best mode of delivery in the case of LVD. However, in neonates with ectopia cordis, a cesarean section is recommended to avoid rupture or external pressure on the heart. 13 In our patient with a prenatal diagnosis of a complex intracardiac anomaly, vaginal delivery was feasible. To confirm LVD postnatally, left ventricular angiography is the gold standard. In our patient, we performed cardiac contrast-enhanced MRI as a noninvasive alternative method ( Video 1 ).

Patients with LVD have a higher risk of ventricular tachyarrhythmia (9.9%), heart failure (6.8%), or embolic events (2.9%). Perioperative mortality is about 7% caused by rupture of the LVD (75% of all patients) and sudden cardiac death (25%). Rupture occurs by an increased systolic blood pressure in the diverticulum. These cases are mostly described in patients younger than 8 years. 6 There is lacking evidence on the relationship between the size of the diverticulum and the risk of complications. This might be the reason for conservative treatment of adults with a small asymptomatic LVD. 9

There are several classifications of LVD. One of them is morphological and classified LVD in a nonapical and apical types. The apical type is more often associated with other cardiac defects or syndromic diseases. 14 Our patient showed an apical type with associated cardiac defects in the absence of associated syndromic features. Malakan Rad et al 8 proposed a novel classification system for different left ventricular outpouchings (LVO) including the LVD, LVA, double-chambered left ventricle, and accessory left ventricle. Furthermore, the authors also developed a grading system for the size of LVO that includes volume, area, and circumference index.

### Surgery

Successful surgical resection of an LVD in a neonate was first described in 1944. 15 16 As the procedure depends on the size and type of the diverticulum, there is no standard surgical management. In the case of an LVD, early elective surgery is recommended to avoid thrombosis, rupture, or tachyarrhythmia. 13 17 18 Most authors recommend resection of the LVD with patch closure especially when the distance to the left ventricle is more than 2 cm. 12 In our case, the diverticulum was small, and there were no other major cardiac malformations; therefore, surgical resection and primary closure were feasible.

## Conclusion

In a neonate with a pulsating supraumbilical mass without sternal cleft or omphalocele, the diagnosis of LVD should be considered. In the case of LVD, early surgical repair is preferred to avoid rupture, thrombosis, or arrhythmia.

## Notes

New Insights & the Importance for the Pediatric Surgeon This case gives new insights into a special and rare case where interdisciplinary work is necessary for success.
